# Feasibility study of first-line chemotherapy using Pemetrexed and Bevacizumab for advanced or recurrent nonsquamous non-small cell lung cancer in elderly patients: TORG1015

**DOI:** 10.1186/s12885-016-2338-6

**Published:** 2016-05-12

**Authors:** Toshiyuki Kozuki, Naoyuki Nogami, Hiromoto Kitajima, Shunichiro Iwasawa, Emiko Sakaida, Yuichi Takiguchi, Satoshi Ikeda, Masahiro Yoshida, Terufumi Kato, Shingo Miyamoto, Kentaro Sakamaki, Tetsu Shinkai, Koshiro Watanabe

**Affiliations:** Department of Thoracic Oncology and Medicine, National Hospital Organization Shikoku Cancer Center, 160 Kou Minamiumemoto, Matsuyama, Ehime 791-0280 Japan; Department of Medical Oncology, Graduate School of Medicine, Chiba University, Chiba, Japan; Department of Respiratory Medicine, Kanagawa Cardiovascular and Respiratory Center, Yokohama, Japan; Department of Oncology, Japanese Red Cross Medical Center, Tokyo, Japan; Department of Biostatistics, Graduate School of Medicine, Yokohama City University, Yokohama, Japan; Thoracic Oncology Research Group, Yokohama, Japan

**Keywords:** Non-small cell lung cancer, Bevacizumab, Pemetrexed, Elderly, Feasibility study

## Abstract

**Background:**

The addition of bevacizumab to cytotoxic agents prolongs survival in patients with nonsquamous non-small cell lung cancer (NSCLC). To date, there is no evidence to suggest that treatment with a cytotoxic agent plus bevacizumab is more effective than a cytotoxic agent alone for nonsquamous NSCLC in elderly patients. We conducted a feasibility study of pemetrexed plus bevacizumab as a first-line treatment for advanced or recurrent nonsquamous NSCLC in elderly patients.

**Methods:**

Major eligibility and exclusion criteria included: chemotherapy-naive status; non-fitness for bolus combination chemotherapy; stage III/IV or relapsed nonsquamous NSCLC; age ≥70; performance status 0–1; absence of brain metastasis; and no history of hemoptysis and thoracic irradiation. Pemetrexed (500 mg/m^2^) and bevacizumab (15 mg/kg) were administered intravenously on day 1, and repeated every 3 weeks thereafter. The primary endpoint was safety, and the secondary endpoints were objective response rate (ORR), progression-free survival (PFS), overall survival (OS), and the percentage of patients who completed ≥3 cycles.

**Results:**

From October 2010 to April 2012, a total of 12 patients were enrolled. No dose-limiting toxicity or treatment-related deaths were observed. Three patients achieved PR, and the ORR was 25 %. The median PFS and OS were 5.4 months (95 % CI 1.1–8.8 months) and 13.6 months (95 % CI 5.3–15.6 months), respectively. Seven of 12 patients (58 %) received ≥3 cycles.

**Conclusions:**

Pemetrexed plus bevacizumab in the treatment of elderly patients with nonsquamous NSCLC was well tolerated and shows promise as first-line treatment.

**Trial registration:**

UMIN Clinical Trial Registry; UMIN000004263. Registered on 25 September, 2010.

## Background

Lung cancer is the leading cause of cancer-related deaths in Japan. With the gradual increase in the elderly population, more than half of lung cancer patients are over 70 years old [1]. Therefore, there is an urgent need to develop a treatment strategy especially tailored for elderly patients with advanced or recurrent non-small cell lung cancer (NSCLC).

Standard treatment of elderly advanced NSCLC patients involves monotherapy with vinorelbine or gemcitabine, however, two phase III trials in Japan showed docetaxel monotherapy to also be suitable for treatment of elderly patients with NSCLC [2, 3].

Pemetrexed is a multi-targeted anti-folate chemotherapeutic agent. Studies showed pemetrexed is an alternative option to third-generation agents, such as gemcitabine or docetaxel, in the treatment of advanced or recurrent NSCLC in a first- or second-line setting [4, 5]. Moreover, monotherapy using pemetrexed showed a favourable anti-tumor effect with mild toxicity [5]. Thus due to its mild toxicity profile, pemetrexed could represent a promising chemotherapeutic agent for nonsquamous non-small cell malignancy especially for elderly patients.

Bevacizumab is a humanized monoclonal antibody against vascular endothelial growth factor (VEGF). A randomized phase III trial showed the addition of bevacizumab to the combination of carboplatin and paclitaxel significantly prolongs the survival for “bevacizumab-fit patients” with advanced or recurrent nonsquamous NSCLC, with a hazard ratio for death of 0.79 [6]. Moreover, a recent randomized phase II trial of Japanese nonsquamous NSCLC patients also reported a similar hazard ratio (0.61) for progression-free survival in patients under 75 years [7]. However, there is no definitive evidence that the addition of bevacizumab to a non-platinum agent monotherapy is safe and superior in terms of efficacy, compared to monotherapy, in the treatment of elderly patients. Therefore, in order to assess the safety of bevacizumab, we conducted a feasibility study of pemetrexed combined with bevacizumab in the treatment of elderly patients with nonsquamous NSCLC.

## Methods

### Study participants

We carried out a multicenter feasibility study of previously untreated elderly patients with nonsquamous NSCLC from nine healthcare institutions. This study was approved by the ethics committee of all participating hospitals (Shikoku Cancer Center, Chiba University Hospital, Kanagawa Cardiovascular and Respiratory Center, Japanese Red Cross Medical Center, Fujisawa City Hospital, Nippon Medical School Chiba Hokusoh Hospital, and Okayama Rosai Hospital). Actual enrolments were performed at four hospitals (Shikoku Cancer Center, Chiba University Hospital, Kanagawa Cardiovascular and Respiratory Center, and Japanese Red Cross Medical Hospital). Written informed consent was obtained from all participating patients. This trial was registered in University Hospital Medical Information Network (UMIN) Clinical Trials Registry (UMIN-CTR), issue number UMIN000004263.

Eligible patients had histologically or cytologically confirmed nonsquamous NSCLC; an Eastern Cooperative Oncology Group (ECOG) performance status (PS) of 0 or 1; measurable lesions as defined by Response Evaluation Criteria in Solid Tumors (RECIST) version 1.1; and no prior chemotherapy (except for uracil and tegafur or epidermal growth factor receptor (EGFR) tyrosine kinase inhibitor). Patients were unfit for bolus administration of cisplatin or combination chemotherapy, and had adequate bone marrow, hepatic, and renal functions (leucocyte counts ≥4000/mm^3^, absolute neutrophil counts ≥2000/mm^3^, platelet counts ≥100,000/mm^3^, haemoglobin ≥9.5 g/dl, serum aspartate aminotransferase (AST) ≤2.5× upper limit of normal (ULN) range, alanine aminotransferase (ALT) ≤2.5× ULN, total bilirubin ≤1.5 mg/dl, serum creatinine ≤1.5 mg/dl, and urinalysis of proteinuria by dipstick (dipstick result ≤(+)). Key exclusion criteria included the presence of brain metastasis, a history of hemoptysis (≥2.5 ml), active infectious disease, massive pleural effusion, pericardial effusion, or abdominal effusion, severe co-morbidity (heart disease, interstitial lung disease, inadequately controlled hypertension, or diabetes mellitus), a history of thoracic irradiation, concomitant malignancy within the last 5 years, coagulation disorders or therapeutic anti-coagulation, gastrointestinal perforation, minor surgery within the last 2 weeks, major surgery within the last 4 weeks, and major surgery with lobectomy or pneumonectomy within the last 8 weeks.

### Study design

Patients were administered intravenously with pemetrexed (500 mg/m^2^) and bevacizumab (15 mg/kg) on day 1, with repeat administration given every 3 weeks thereafter—defined as level 0. The planned number of patients enrolled in this trial consisted of a minimum of 12 to a maximum of 24 patients. If dose-limiting toxicities (DLTs) were observed in ≥4 out of six patients or ≥6 out of 12 patients at level 0, we evaluated the patients as level −1 when pemetrexed dosage was reduced to 375 mg/m^2^. If <6 out of 12 patients experienced DLTs at each level, this dose level was considered to be feasible. Treatment was repeated for a total of 6 cycles, although if toxicity was acceptable, treatment was continued until disease progression.

DLTs were evaluated in the first cycle of chemotherapy and defined according to the National Cancer Institute Common Terminology Criteria for Adverse Event (NCI CTCAE) version 4.03: grade 4 neutropenia lasting for ≥4 days; febrile neutropenia; grade 4 thrombocytopenia; and grades 3–5 nonhaematological toxicities (except for nausea, hyponatremia, weight loss, anorexia, or hypertension).

The primary endpoint of this study was safety, and the secondary endpoints were the progression-free survival (PFS), overall survival (OS), objective response ratio (ORR) according to RECIST version 1.1, and the completion ratio of three cycles or more.

### Statistical analysis

Survival curves were estimated using the Kaplan–Meier method. PFS was defined as starting from the date of enrolment to the first documented date of disease progression or last contact. OS was defined as starting from the date of enrolment to the date of death from any causes, or last contact.

## Results

### Patient characteristics

This study was carried out between October 2010 and April 2012. A total of 12 previously untreated elderly patients (≥70 years old) with nonsquamous NSCLC were enrolled. Patients’ baseline characteristics are shown in Table 1. Half of patients were female, ECOG PS 0, or never-smokers. Median age was 78 years (range 72–81), and 11 (92 %) of 12 patients were 75 years or more. Histology confirmed adenocarcinoma in all cases. Eleven patients were assessed for the activating *EGFR* mutation status using commercially available highly-sensitive methods such as the peptide nucleic acid-locked nucleic acid (PNA-LNA) PCR-clamp methods, cycleave PCR, PCR-Invader, or Scorpion ARMS methods. We found two patients (18 %) who harboured the *EGFR* mutation in exon 21.

**Table 1:** Patients characteristics

Male/Female	6/6
Age median (range)	78 (72–81)
<75/≥75	1/11
ECOG PS 0/1	6/6
Smoking (Current/former/never)	1/5/6
Stage IIIB/IV/Recurrent disease	2/8/2
Histology (adenocarcinoma/others)	12/0
Activating EGFR mutation (No/Yes/Unknown)	9/2/1

*ECOG* Eastern cooperative Oncology Group, *PS* performance status

### Treatment delivery

The median administration cycle was 4.5 cycles (range 1–8). Seven patients (58 %) completed ≥3 cycles. No patient needed dose modification during their treatment. Reasons for treatment discontinuation included the following: 5 of 12 (42 %) patients requested termination of treatment (three cases may have been related to adverse events; one case was geographically related whereby it was difficult for the patient to make regular travel arrangements to the hospital; one case was related to the exacerbation of co-morbidity); three cases (25 %) were due to disease progression; 2 (17 %) cases were due to toxicities (stroke and sigmoid colon perforation, respectively). The details which three patients who asked for stopping the treatment might be related to the adverse events were followings; One experienced grade 2 anorexia, nausea, and fatigue during 2 cycles of treatment, another experienced grade 2 stomatitis and grade 1 anorexia and urticaria during 8 cycles of treatment, the other experienced grade 1 anorexia and dosing once only.

Six patients experienced treatment delays due to physicians’ or patients’ conveniences, although all had their treatment started within 1 week of the scheduled date.

### Safety

DLTs were not noted in this study. Grade 3 adverse events included leukopenia (25 %), neutropenia (25 %), and hypertension (8 %). Grade 2 or lower adverse events observed in the first cycle included anaemia, thrombocytopenia, gastritis, and stroke (Table 2). Grade 3 or higher adverse events or those which occurred at >10 % or might be related to discontinuation in the entire treatment are shown in Table 3. Grade 4–5 adverse events were not found in any cycle. Grade 3 adverse events reported in any cycle included leukopenia (25 %), neutropenia (25 %), anaemia (8 %), thrombocytopenia (8 %), febrile neutropenia (8 %), fatigue (8 %), anorexia (8 %), nausea (8 %), perforation (colon) (8 %), and hypertension (8 %). All adverse events in this study were already known and predictable for the safety profiles of pemetrexed or bevacizumab. Reported toxicities were mild and manageable.

**Table 2:** Adverse events in 1^st^ cycle

*N* = 12	CTC-AE grade (Ver. 4.03)				
	1	2	3	4	3/4 (%)
Leukopenia	2	2	3	0	3 (25 %)
Neutropenia	1	3	3	0	3 (25 %)
Anemia	1	1	0	0	0 (0 %)
Thrombocytopenia	3	1	0	0	0 (0 %)
Gastritis	0	1	0	0	0 (0 %)
Stroke	0	1	0	0	0 (0 %)

*CTC-AE* common terminology criteria for adverse events

**Table 3:** Adverse events (All cycles)

*N* = 12	CTC-AE grade (Ver. 4.03)				
	1	2	3	4	3/4 (%)
Leukopenia	1	3	3	0	3 (25 %)
Neutropenia	2	3	3	0	3 (25 %)
Anemia	4	1	1	0	1 (8 %)
Thrombocytopenia	4	0	1	0	1 (8 %)
Febrile Neutropenia	0	0	1	0	1 (8 %)
Hypoalbuminemia	7	1	0	0	0 (0 %)
T-Bil elevation	1	1	0	0	0 (0 %)
AST elevation	6	0	0	0	0 (0 %)
ALT elevation	4	0	0	0	0 (0 %)
ALP elevation	4	0	0	0	0 (0 %)
Cre elevation	3	0	0	0	0 (0 %)
Hyperkalemia	2	0	0	0	0 (0 %)
Hypercalcemia	2	0	0	0	0 (0 %)
Hyponatremia	2	0	0	0	0 (0 %)
Fatigue	3	1	1	0	1 (8 %)
Anorexia	4	3	1	0	1 (8 %)
Weight loss	1	1	0	0	0 (0 %)
Nausea	1	2	1	0	1 (8 %)
Vomiting	2	0	0	0	0 (0 %)
Constipation	2	4	0	0	0 (0 %)
Perforation (colon)	0	0	1	0	1 (8 %)
Hypertension	1	2	0	0	0 (0 %)
Epistaxis	3	0	0	0	0 (0 %)
Stroke	0	1	0	0	0 (0 %)
Urticaria	1	1	0	0	0 (0 %)
Pruritus	2	0	0	0	0 (0 %)
Pneumonitis	2	0	0	0	0 (0 %)

*CTC-AE* common terminology criteria for adverse events

### Treatment efficacy

**Fig. 1:**
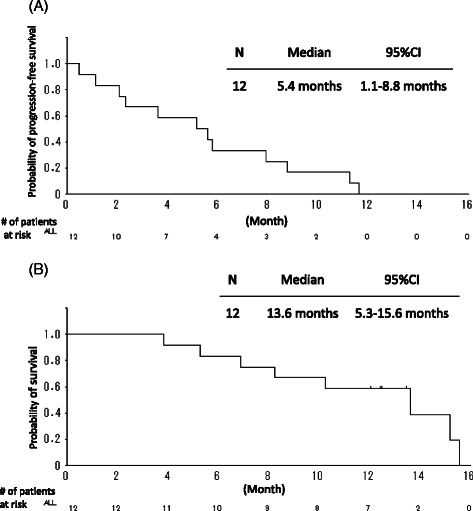
Survival curves. **a** Progression-free survival (PFS) and (**b**) overall survival (OS). At a median follow-up time of 12.2 months (range, 3.8–15.4 months), the median PFS and OS times were 5.4 and 13.6 months, respectively

Three of 12 patients achieved partial response (PR), six patients stable disease (SD), and 3 progressive disease (PD). No patient achieved complete response (CR). The best ORR was 25 % (95 % confidence interval (CI) 5.5–57.2 %). The disease control rate (DCR; CR + PR + SD) was 75 % (95 % CI 42.8–94.5 %). Of the 9 patients that had wild-type *EGFR*, three achieved PR and the best ORR was 33 %. The best ORR in PS-0 patients was 50 %, in contrast, no PS-1 patient achieved PR. The median follow-up duration was 12.2 months (range, 3.8–15.4 months). The median PFS and OS were 5.4 months (95 % CI 1.1–8.8 months) and 13.6 months (95 % CI, 5.3–15.6 months), respectively (Fig. 1). The 1-year survival rate was 58 % (95 % CI 27–80 %).

## Discussion

This is the first study of combination treatment with pemetrexed and bevacizumab in Japanese chemotherapy-naive elderly patients with advanced or recurrent nonsquamous NSCLC. None of the 12 study patients experienced DLTs, and reported toxicities were mild. Therefore, we conclude the combination of pemetrexed 500 mg/m^2^ plus bevacizumab 15 mg/kg, both given on day 1 and repeated every 3 weeks, is feasible as first-line therapy for elderly patients (≥70) with nonsquamous NSCLC.

The addition of bevacizumab to the platinum doublet prolonged PFS and enhanced the antitumor activity [6, 8]. The addition of bevacizumab to carboplatin and paclitaxel significantly improved the OS in ECOG4599 [6]. A subset analysis of elderly patients (age ≥70 years) failed to show a survival benefit in ECOG4599, while reporting more toxicities and a higher incidence of treatment-related death (TRD) of 6.3 and 2.6 % in the elderly and younger cohort, respectively [9]. In our study, we reported no occurrence of TRD using the combination therapy of bevacizumab with pemetrexed. The SAiL study, a phase IV bevacizumab cohort study, described a slightly higher rate of severe adverse events in elderly patients (>65 years; 45.3 %), compared with younger patients (≤65 years; 34.7 %) [10]. Furthermore, the incidences of adverse events of special interest (AESI) (including hypertension; proteinuria; wound healing complications; gastrointestinal perforations; arterial and venous thromboembolic events; hemoptysis; central nervous system bleeding; other haemorrhages; and congestive heart failure) for bevacizumab were 70.8 and 68.3 % in the elderly and younger cohorts, respectively. In addition, the incidences of grade ≥3 gastrointestinal perforation were 1.4 and 0.8 % for the elderly (>70 years) and younger patients (≤70 years), respectively [10]. Another group also conducted a prospective cohort study to assess the toxicity of bevacizumab plus chemotherapy for elderly patients aged ≥65 in a practical setting [11]. The frequency of haematologic toxicities, nonhaematologic toxicities, hospitalizations, dose reduction, dose delay, and discontinuation of treatment were not significantly associated with bevacizumab plus chemotherapy [9]. However, the addition of bevacizumab was associated with a high frequency of grade 3–5 toxicities in a multivariate analysis, with an odds ratio of 2.86 (95 % CI 1.06–7.70; *p* = 0.038). Nearly 70 % of patients received the combination chemotherapy, which was found to be generally more toxic than monotherapy, in this analysis [9].

In our study, five patients experienced grade 3 toxicities but none reported grade 4–5 toxicities following the treatment combination of pemetrexed and bevacizumab. The frequency of AESI was also comparable (Table 4). We observed grade 3 intestinal perforation in one patient (8 %) who underwent sigmoidectomy for a perforated diverticulum in the sigmoid colon. Although this patient had received 6 cycles of bevacizumab previously, the surgical operation was performed successfully without any major complications. Overall the reported toxicities of pemetrexed were mild and therefore it was concluded that the addition of bevacizumab to pemetrexed was acceptable despite the occurrence of slightly more toxicities suggesting this combination as a feasible treatment.

**Table 4:** Incidence of adverse events of special interest for bevacizumab

*N* = 12	CTC-AE grade (Ver. 4.03)				
	1	2	3	4	Total (%)
Hypertension	2	2	0	0	4 (33 %)
Epistaxis	3	0	0	0	3 (25 %)
Throat Bleeding	1	0	0	0	1 (8 %)
Stroke	0	1	0	0	1 (8 %)
Perforation (colon)	0	0	1	0	1 (8 %)
Proteinuria	1	0	0	0	1 (8 %)
Bronchopulmonary bleeding	0	0	0	0	0 (0 %)
AESI (Most severe grade)	4	2	1	0	7 (58 %)

*AESI* adverse events of special interest, *CTC-AE* common terminology criteria for adverse events

For over a decade, vinorelbine or gemcitabine monotherapy has been shown to prolong survival in chemotherapy-naive elderly patients with advanced NSCLC, and these were considered as the standard treatment for elderly patients with NSCLC [12, 13]. Quoix and colleagues described carboplatin and weekly paclitaxel showed significant survival benefit, compared to vinorelbine monotherapy despite increased toxicities [14]. In Japan, Kudoh and colleagues conducted the phase III trial where docetaxel was compared with vinorelbine in elderly patients and showed no significant difference in the OS [2]. However, docetaxel significantly improved PFS, ORR, and disease-related symptoms therefore docetaxel is considered as the standard treatment in Japan. Recently, a phase III trial of weekly docetaxel plus cisplatin in comparison with docetaxel monotherapy in elderly patients with NSCLC was terminated in the preplanned interim analysis as there was no significant difference in the combination therapy over the monotherapy [3]. Thus, docetaxel monotherapy is still considered as the state-of-the-art treatment for chemotherapy-naive elderly patients with NSCLC in Japan. The efficacy of pemetrexed monotherapy was found to be comparable with docetaxel as second-line treatment for NSCLC [5]. Pemetrexed monotherapy is also promising for chemotherapy-naive elderly patients with advanced NSCLC and could be a substitute for docetaxel monotherapy. A recent phase II study of pemetrexed monotherapy for chemotherapy-naive elderly patients with nonsquamous NSCLC failed to meet its primary endpoint as the ORR [15]. However, the ORR was 25 %, and the median PFS and OS were 3.3 and 17.5 months, respectively. Whilst the median OS in our study was 13.6 months, the ORR and PFS were comparable to those from previous reports [2, 3]. Our study of bevacizumab plus pemetrexed showed the ORR was similar to that with pemetrexed monotherapy. However, the median PFS with the combination therapy was better than with monotherapy and consistent with the study of the use of first-line platinum doublet for NSCLC [16]. It is difficult to evaluate the pemetrexed with bevacizumab combination for elderly NSCLC patients exactly from this study. The addition of bevacizumab is likely to enhance the clinical efficacy of monotherapy in elderly patients with nonsquamous NSCLC without the increased risk of severe toxicities.

## Conclusion

Combination chemotherapy consisting of pemetrexed (500 mg/m^2^ on day 1, Q3w) and bevacizumab (15 mg/kg on day1, Q3w) for elderly patients with advanced or recurrent nonsquamous NSCLC as the first-line treatment was well tolerated, with favourable antitumor activity. Further studies on the treatment of NSCLC in elderly patients are warranted to determine the efficacy of the combination of bevacizumab plus pemetrexed.

### Ethics approval

This study was approval by the ethics committee of Shikoku Cancer Center, Chiba University Hospital, Kanagawa Cardiovascular and Respiratory Center, Japanese Red Cross Medical Center, Fujisawa City Hospital, Nippon Medical School Chiba Hokusoh Hospital, and Okayama Rosai Hospital before participation in this study.

### Consent for publication

The consents were obtained from all participating patients.

### Availability of data and materials

The datasets supporting the conclusion of this article is included within article.

## Abbreviations

NSCLC: non-small cell lung cancer
TORG: Thoracic Oncology Research Group
PFS: progression-free survival
OS: overall survival
PR: partial response
ORR: objective response rate
UMIN: University Hospital Medical Information Network
EGF: epidermal growth factor
CTR: Clinical Trials Registry
ECOG: Eastern Cooperative Oncology Group
PS: performance status
RECIST: response evaluation criteria in solid tumors
EGFR: epidermal growth factor receptor
AST: aspartate aminotransferase
ALT: alanine aminotransferase
ULN: upper limit of normal
DLT: dose-limiting toxicity
NCI CTCAE: National Cancer Institute Common Terminology Criteria for Adverse Events
PCR: polymerase chain reaction
PNA-LNA: peptide nucleic acid-locked nucleic acid
ARMS: amplification refractory mutation system
SD: stable disease
CR: complete response
CI: confidence interval
DCR: disease control rate
TRD: treatment-related death
AESI: adverse events of special interest
